# Exploring the growing phase forest musk deer (*Moschus berezovskii*) dietary energy requirements based on growth performance and gut microbiota analysis

**DOI:** 10.1128/spectrum.02352-24

**Published:** 2025-01-28

**Authors:** Ruiguang Gong, Xiaocao Di, Xinxin Ping, Haodong Han, Bing Song, Shuhui Wang, Xianggui Dong, Zhan Jun Ren

**Affiliations:** 1 College of Animal Science and Technology, Northwest A&F University 546344 , Yangling, China; University of Valencia, Paterna, Valencia, Spain

**Keywords:** *Moschus berezovskii*, digestible energy, growth performance, fecal microbiota, 16S rRNA

## Abstract

The forest musk deer (FMD) farming industry is currently experiencing rapid growth, yet the dietary energy requirements for FMD remain unclear. Therefore, we explored the optimal dietary digestible energy (DE) for growing phase FMD by providing three diets with different DE levels and analyzing changes in gut microbiota. A total of 20 six-month-old FMD were used in a 62-day trial. Animals were fed either traditional feed (CON, 10.38 MJ/kg DE) or pelleted diets with low (L, 8.87 MJ/kg), medium (M, 10.38 MJ/kg), or high (H, 11.86 MJ/kg) DE levels. The results showed that feeding pelleted diets significantly reduced the incidence of diarrhea (*P* < 0.05). The average daily gain in the H group was significantly higher than in the M group, and the M group was higher than both the L and CON groups (*P* < 0.05). Additionally, the H group showed the highest nutrient digestibility and the greatest increase in body measurements (*P* < 0.05). Analysis of fecal microbiota revealed that α-diversity and the relative abundance of *Firmicutes* increased with higher dietary energy levels, while the relative abundance of *Bacteroidetes* decreased. Beneficial bacteria such as *Akkermansia* and *Lachnospira* were significantly enriched in the H group. In conclusion, our findings suggest that pelleted diets are suitable for growing phase FMD, with an optimal dietary DE level of 11.86 MJ/kg.

**IMPORTANCE:**

This study underscores the significance of identifying the optimal dietary digestible energy (DE) for growing forest musk deer (FMD). Pelleted diets with a DE level of 11.86 MJ/kg enhanced growth performance, nutrient digestibility, and gut health, while reducing diarrhea and enriching beneficial gut bacteria, offering valuable insights for improving FMD farming practices.

## INTRODUCTION

The forest musk deer (FMD) (*Moschus berezovskii*) secretes musk, which possesses high medicinal and economic value, leading to extensive poaching in the past. To protect the species and address the supply-demand imbalance in the market, China initiated the artificial breeding of FMD in the early 1950s (1). Currently, captive FMDs are primarily fed green succulent feed, along with fresh and dried leaves, supplemented with cornmeal, soybean meal, and wheat bran (2). However, green succulent feed is prone to spoilage in summer and freezing in winter (3), and the collection and drying of tree leaves require significant labor, resulting in high feed costs for FMD farms.

Young FMDs are typically born between May and June each year (4). By December, they are usually around 6 months old, corresponding to a juvenile stage in humans. At this age, they are fully weaned and capable of being fed solely with formulated feed. However, they have not yet reached either physical or sexual maturity, and their bodily functions remain in a phase of rapid growth and development, leading to high nutritional demands. Therefore, the quality of the diet during this growth stage is crucial, significantly influencing their subsequent health status, growth performance, and reproductive capacity (5). Currently, feeding practices for young musk deer lack standardized dietary plans and precise nutritional requirements (6). Additionally, during autumn and winter, lower temperatures increase energy expenditure. The traditional use of high-moisture root and tuber feeds may result in insufficient energy intake (7). This often leads to slow development and poor body condition among young musk deer, thereby constraining their growth, genetic potential, and the overall development of the musk deer farming industry.

The gut microbiota plays a crucial role in host growth and health (8), and the composition and nutrient content of the diet directly influence the structure and function of the gut microbial community (9). In FMD, analyzing changes in fecal microbiota is an important method for monitoring their health status (10, 11).

This study investigates the effects of total mixed pellet feed with different digestible energy (DE) levels on diarrhea rate, growth performance, nutrient digestibility, and fecal microbiota in growing FMD, providing data to inform nutritional requirements and feeding standards for the species.

## MATERIALS AND METHODS

### Animals and diets

Twenty FMD (6 months old, with an average body weight of 4.90 ± 0.15 kg) were selected and fed equal portions at 9:00 a.m. and 4:00 p.m. daily. All animals were housed individually, with *ad libitum* access to feed and water. The pre-trial period lasted 8 days, followed by a formal experimental period of 62 days.

Based on traditional feeding methods, the nutritional composition of the dried feed ingredients was determined, and the feed intake of the FMD was recorded. Subsequently, three total mixed rations with different DE levels were formulated. The 20 musk deer were randomly assigned to four groups, with five replicates per group, and one animal per replicate. The CON group was fed according to traditional methods, with the diet composition and weight ratios as follows: fresh carrots:fresh pumpkins:cornmeal:soybean meal:dried leaves = 9:9:1:1:2, with a DE of 10.38 MJ/kg on a dry matter basis. The L and M groups were provided with total mixed pellet diets containing 8.87 and 10.38 MJ/kg DE, respectively, while the H group received a diet with 11.86 MJ/kg DE, based on Zhang et al. in goats (12). All feed ingredients were ground and pelleted using a ring die pellet mill (compression ratio 1:8, pore diameter 4.0 mm) into pellets with a length of 8.0–15.0 mm. The composition of the diets is shown in Table 1.

**TABLE 1:** Ingredients and nutrient composition of diets

Item	Diet		
	L	M	H
Alfalfa hay	51.25	46.82	40.88
Soybean meal	5.31	5.91	5.84
Mulberry branch bark powder	2.44	4.22	5.33
Broussonetia papyrifera silage	2.58	4.81	5.00
Beet pulp	0.00	2.03	4.82
Wheat middling	0.00	0.00	4.93
Wheat bran	12.60	6.20	0.00
Corn	20.32	24.51	24.40
Soybean oil	0.50	0.50	0.80
Vitamin mineral premix^a^	5.00	5.00	5.00
Nutrient composition, % of DM^b^			
Digestible energy, MJ/kg	8.87	10.38	11.86
CP^c^	13.33	13.33	13.34
NDF^d^	26.41	26.42	26.39
CF^e^	13.10	12.80	11.91
Lysine	1.70	1.70	1.70
Methionine + cystine	0.60	0.60	0.60
Ca	0.84	0.85	0.85
P	0.23	0.23	0.23

^a^ Provided per kilogram diet: 8,000 IU of vitamin A; 500 IU of vitamin D_3_; 10 IU of vitamin E; 2 mg of vitamin K_3_; 50 mg of Fe (as ferrous sulfate); 4 mg of Cu (as copper sulfate); 2 mg of Mn (as manganese sulfate); 50 mg of Zn (as zinc sulfate); 0.2 mg of I (as KI); and 0.2 mg of Se (as Na_2_SeO_3_).

^b^ Dry matter.

^c^ Crude protein.

^d^ Neutral detergent fiber.

^e^ Crude fiber.

### Calculation of diarrhea rate

From the start of the experimental period until the end of the trial, the fecal morphology and color of FMDs were observed daily. Diarrhea was diagnosed when feces were thin, unable to form pellets, or contained mucus, pus, or other abnormal substances.

The diarrhea incidence rate was calculated using the following formula:

Diarrhea incidence rate = (Number of animals with diarrhea × Number of days with diarrhea)/(Total number of animals × Total observation days).

### Measurement and sampling

At the beginning and end of the experiment, after an overnight fasting period (12 h), withers height, body length, body oblique length, chest girth, and body weight were measured. The average daily feed intake (ADFI), average daily gain (ADG), and feed conversion ratio (FCR) were also calculated.

Withers height: Measured as the vertical distance from the top of the withers to the ground using a measuring stick.

Body length: Measured as the horizontal distance from the shoulder joint to the rear edge of the ischium using a measuring tape and a stick.

Body oblique length: Measured as the distance from the most anterior point of the humeral tubercle to the ischial tuberosity using a measuring tape.

Chest girth: Measured as the circumference of the body at the rear edge of the scapula using a measuring tape.

The digestibility of dietary nutrients was determined using the total fecal collection method. From days 55 to 61 of the experiment, feces and urine of FMDs were collected daily before feeding in the morning. To prevent ammonia nitrogen loss, 10% tartaric acid solution was added to the fecal samples, which were stored at −20°C. On the final day of the experiment, fresh fecal samples were collected immediately after defecation, rapidly frozen in liquid nitrogen, and stored at −80°C for further analysis.

### Nutrient composition analysis

All feed and fecal samples were ground and passed through a 1 mm sieve for triplicate measurements. The dry matter (DM), crude protein (CP), neutral detergent fiber (NDF), and acid detergent fiber (ADF) content were analyzed. The apparent total tract digestibility of DM, CP, NDF, and ADF was determined according to the method previously described by Williams et al. (13). Cornmeal, soybean meal, carrot, pumpkin, dry leaves and fecal samples were analyzed for gross energy using an isoperibol bomb calorimeter (ATC 300A, Zeal Instruments, Hangzhou, Zhejiang, China).

### 16S rDNA sequencing

The total genomic DNA of fecal bacteria was extracted using DNA Kit (Code no. 9763, Takara Bio Inc., China) according to the manufacturer’s instructions. The genomic DNA was used as a template for PCR amplification. The V4-V5 hypervariable regions of the 16S rDNA were amplified with the primer pair 515F/806R (515F: 5′-GTGNCAGCMGCCGCGGTAA-3′, 806R: 5′-GGACTACHVGGGTWTCTAAT-3′). About 10 µL mixture containing 40 ng of template DNA, primers, buffer, Taq polymerase and ddH_2_O. The following thermal cycling conditions were used: initial denaturation of 5 min at 95°C, 25 cycles of denaturation at 95°C for 30 s, annealing at 50°C for 30 s, extension at 72°C for 40 s, and a final extension at 72°C for 40 s, and a final extension of almost full-length 16S rRNA gene was purified by agarose gel and ligated into TA cloning kit of TOP 10 (Invitrogen, USA). Sequencing of the 16S rDNA gene was conducted on the Illumina HiSeq X platform, followed by library construction. Sequences were quality-filtered and clustered into operational taxonomic units (OTUs) at 97.0% similarity (14).

Usearch v10.0.240 was used to cluster CCSs at 97% similarity level, obtain OTUs. Venn diagram was drawn with Origin 2021. Conduct taxonomic annotation of the OTUs based on Silva 132 (bacteria) (15) taxonomy databases. Query sequences were blasted against the reference database using the classify-consensus-blast methods, and the nonmatched sequences were further classified by classify-sklearn. Data on the community composition of each sample were obtained at various classification levels (phylum, class, order, family, genus, and species). The alpha diversity index of each sample was evaluated using QIIME2 software (16). Principal coordinate analysis (PCoA) was performed using the mixOmics package in R (v3.2.1) based on the Bray-Curtis distances; non-metric multidimensional scaling was performed using the R package “Vegan.” Microbiota-based biomarker discoveries were performed with linear discriminant analysis effect size (LEfSe) using the online server (https://huttenhower.sph.harvard.edu/galaxy/).

### Statistical analysis

All data are expressed as the mean ± standard error and plotted using GraphPad Prism (version 9.0) software. SPSS software (SPSS 22.0, SPSS Inc.) was used for statistical analysis of the data, and analysis of variance and least-significant difference mean multiple comparison test were used to evaluate the significance of the differences for each parameter of the samples (*P* <  0.05).

## RESULTS

### Effects of diets with different DE levels on the diarrhea rate in growing phase FMD

**Fig 1:**
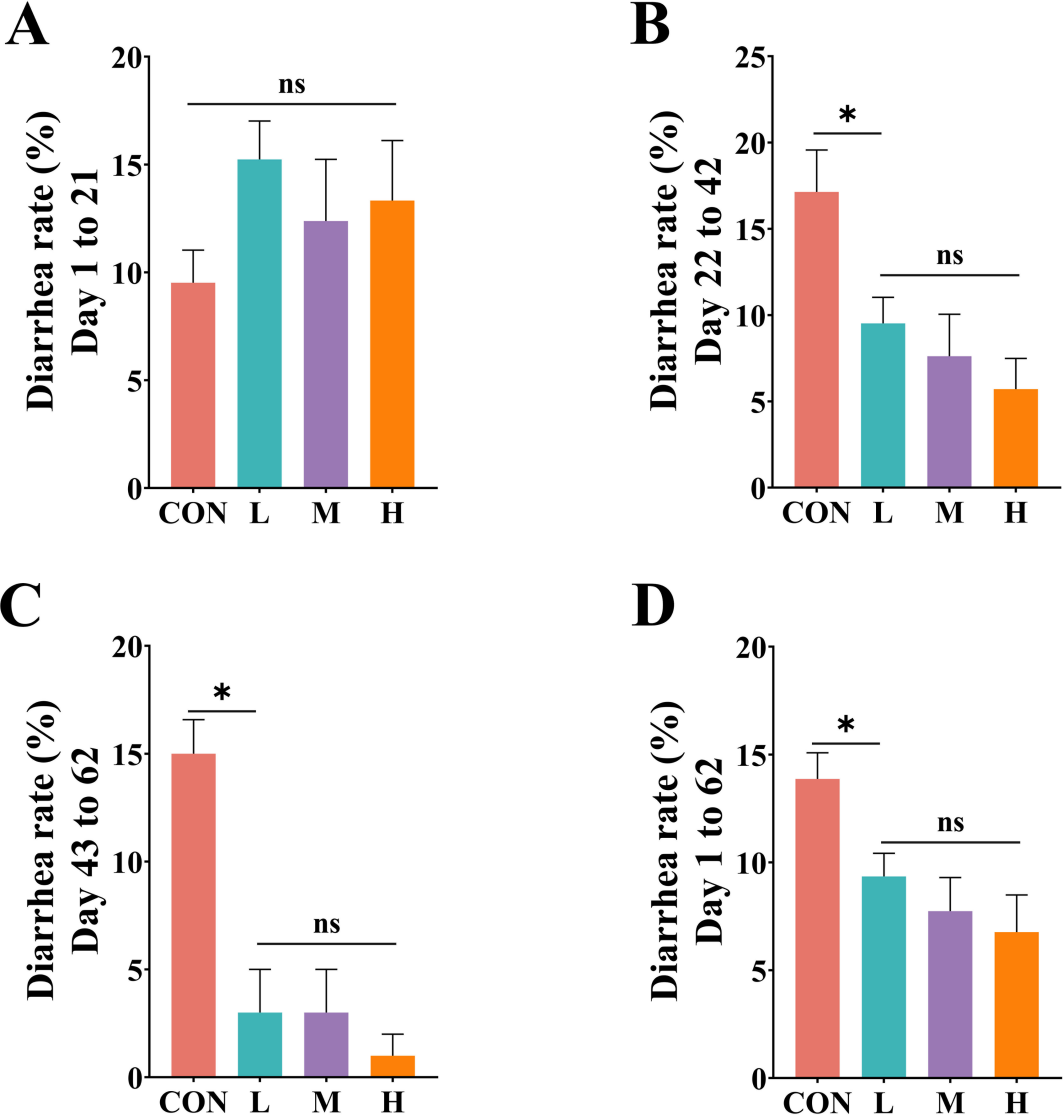
Effects of diets with different digestible energy levels on the diarrhea rate in growing phase forest musk deer (FMD). (**A–D**) Diarrhea rates of FMD during days 1–21, 22–42, 43–62, and the entire trial period. CON, traditional feeding group; L, M, and H represent groups fed pelleted diets with 8.87, 10.38, and 11.86 MJ/kg digestible energy, respectively. *P < 0.05.

The most important criterion for evaluating feed is its safety for consumption. As shown in Fig. 1A, during days 1–21, the diarrhea rate in the CON group was lower than in the other groups, but the difference was not significant (*P* > 0.05). As the experiment progressed, during days 22–42, 43–62, and the overall experimental period, the diarrhea rate in the CON group was significantly higher than in the other three groups (*P* < 0.05) (Fig. 1B, C and D).

### Effects of diets with different DE levels on feed intake, growth performance, and body measurements in growing phase FMD

**Fig 2:**
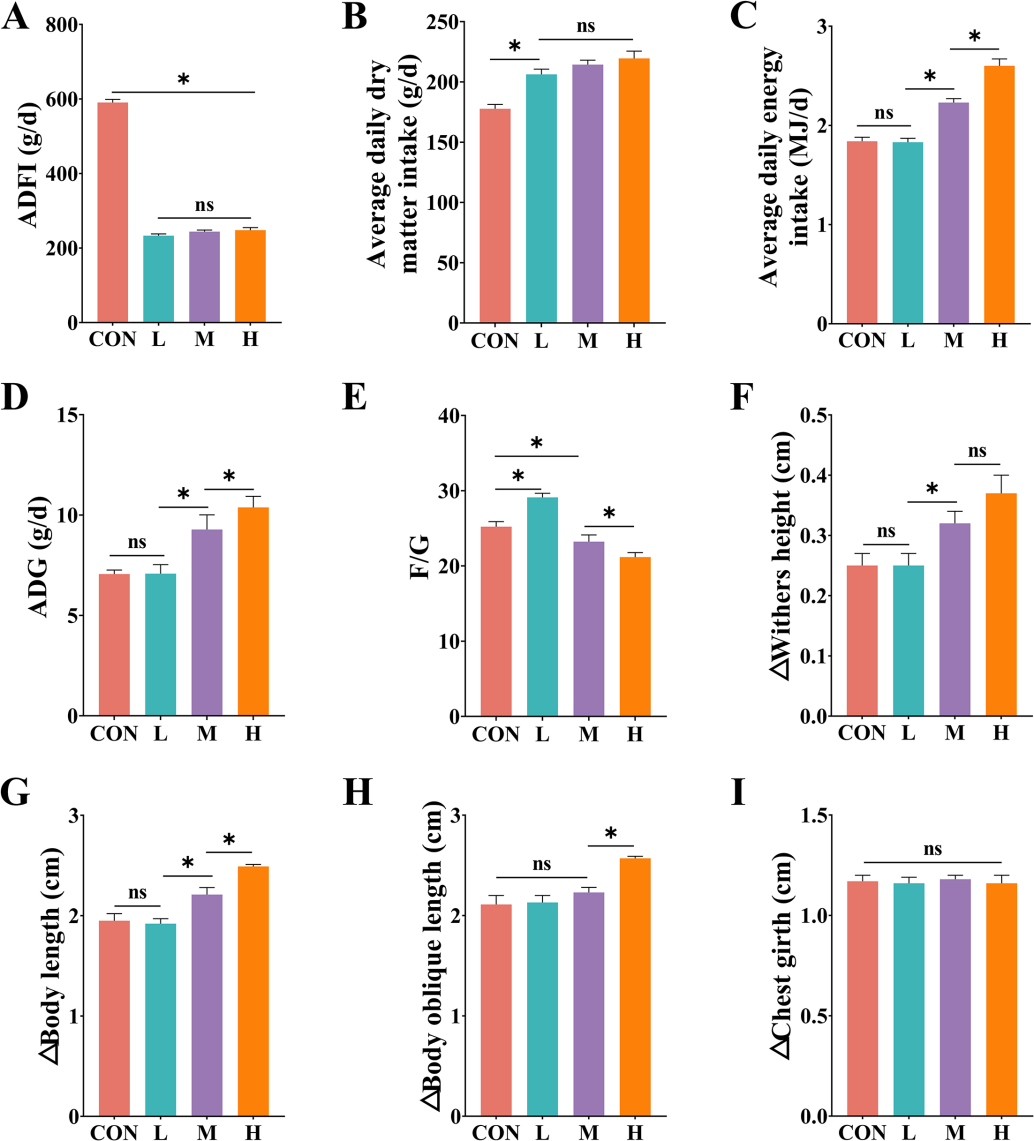
(**A–E**) Effects of diets with different digestible energy levels on feed intake and growth performance in growing phase FMD. ADFI, average daily feed intake; ADG, average daily gain; F/G, feed-to-gain ratio. (**F–I**) Effects of diets with different digestible energy levels on body measurements in growing phase FMD. △Chest girth, △Withers height, △Body length, △Body oblique length = Final body measurements − Initial body measurements.

In the CON group, fed by traditional methods, the ADFI was significantly higher than in the pellet-fed groups due to the high moisture content of carrots and pumpkins in the diet, while the average daily dry matter intake was significantly lower than in the other three groups (*P* < 0.05) ( Fig. 2A and B). Moreover, the H group had the highest average daily energy intake, followed by the M group (*P* < 0.05), with no significant difference between the CON and L groups (Fig. 2C). The ADG in the H group was significantly higher than in the M group, and the M group was significantly higher than in the L and CON groups (*P* < 0.05) (Fig. 2D). The FCR decreased progressively across the L, CON, M, and H groups, with significant differences between the groups (*P* < 0.05) (Fig. 2E).

During the experiment, changes in chest girth among the groups were not significant (*P* > 0.05) (Fig. 2F). However, the withers height in the H and M groups increased significantly compared to the L and CON groups (*P* < 0.05) (Fig. 2G). Changes in body length progressively decreased in the H, M, and L groups (*P* < 0.05), while there was no significant difference between the L and CON groups (*P* > 0.05) (Fig. 2H). Changes in body oblique length were not significantly different between the CON, L, and M groups (*P* > 0.05), but all were significantly lower than in the H group (*P* < 0.05) (Fig. 2I).

### Effects of diets with different DE levels on nutrient digestibility in growing phase FMD

As shown in Table 2, for DM digestibility, the CON group had higher digestibility than the L group but lower than the H group (*P* < 0.05), while the difference with the M group was not significant (*P* > 0.05). The L group had significantly lower CP digestibility compared to the other three groups. NDF digestibility was significantly higher in the H and CON groups than in the L and M groups (*P* < 0.05). The digestibility of ADF was similar among the groups, with no significant differences (*P* > 0.05).

**TABLE 2:** Effects of diets with different digestible energy levels on nutrient digestibility in growing phase FMD^*a*^

Item	CON	L	M	H
DM	60.07 ± 0.56^b^	58.81 ± 0.58^c^	60.02 ± 0.60^bc^	63.44 ± 0.77^a^
CP	54.56 ± 0.51^a^	49.33 ± 0.55^b^	53.88 ± 0.61^a^	54.24 ± 0.78^a^
NDF	54.54 ± 0.55^a^	49.25 ± 0.45^b^	50.98 ± 0.74^b^	54.80 ± 0.70^a^
ADF^*b*^	50.16 ± 0.87	48.33 ± 1.05	50.34 ± 1.00	49.13 ± 1.04

^*a*^ Within rows, values with different superscript letters differ significantly (*P* < 0.05).

^*b*^ Acid detergent fiber.

### Effects of diets with different DE levels on the fecal microbiota of growing phase FMD

#### Alpha diversity and taxonomic analysis based on OTUs

**Fig 3:**
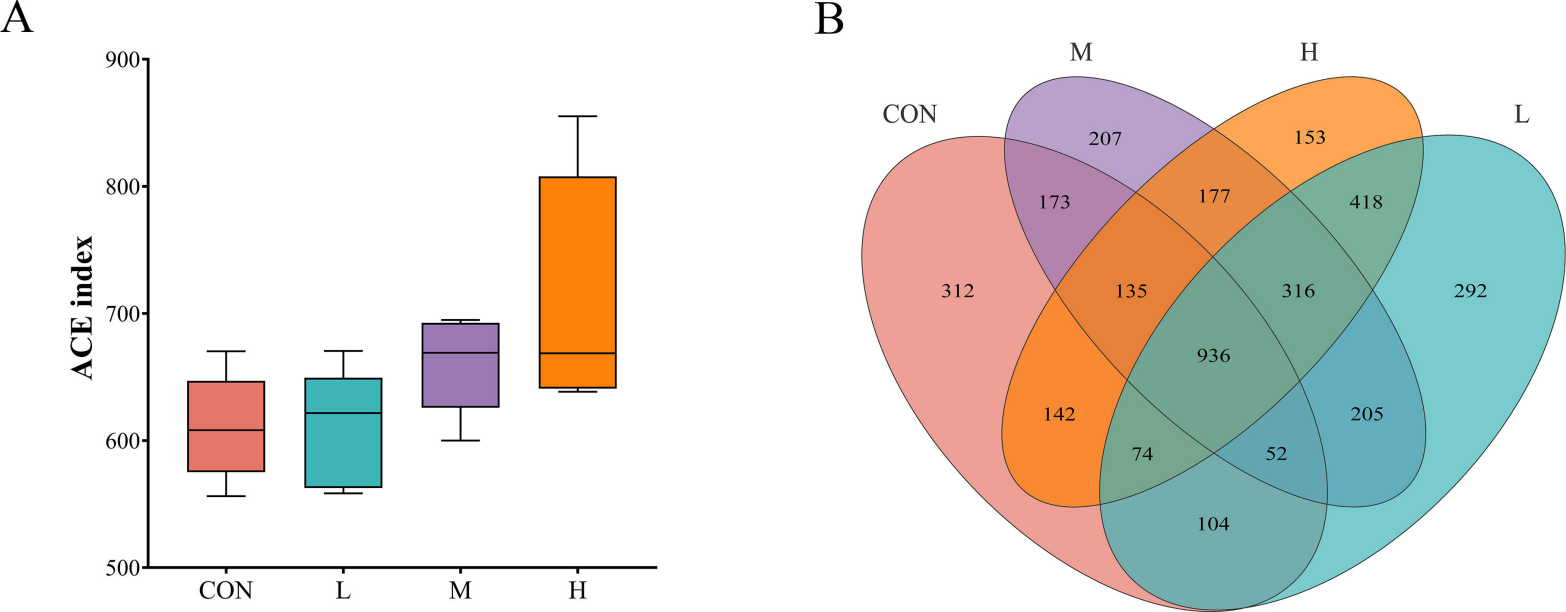
Abundance-based coverage estimator, ACE index (**A**) and venn diagram (**B**) of fecal microbiota in growing phase FMD fed diets with different digestible energy levels.

As shown in Fig. 3A, the ACE index of the fecal microbiota in the CON, L, M, and H groups showed an increasing trend based on OTU analysis. The number of OTUs in each group was 1,928, 2,201, 2,351, and 2,397, respectively, with 936 OTUs shared among the four groups (Fig. 3B).

**Fig 4:**
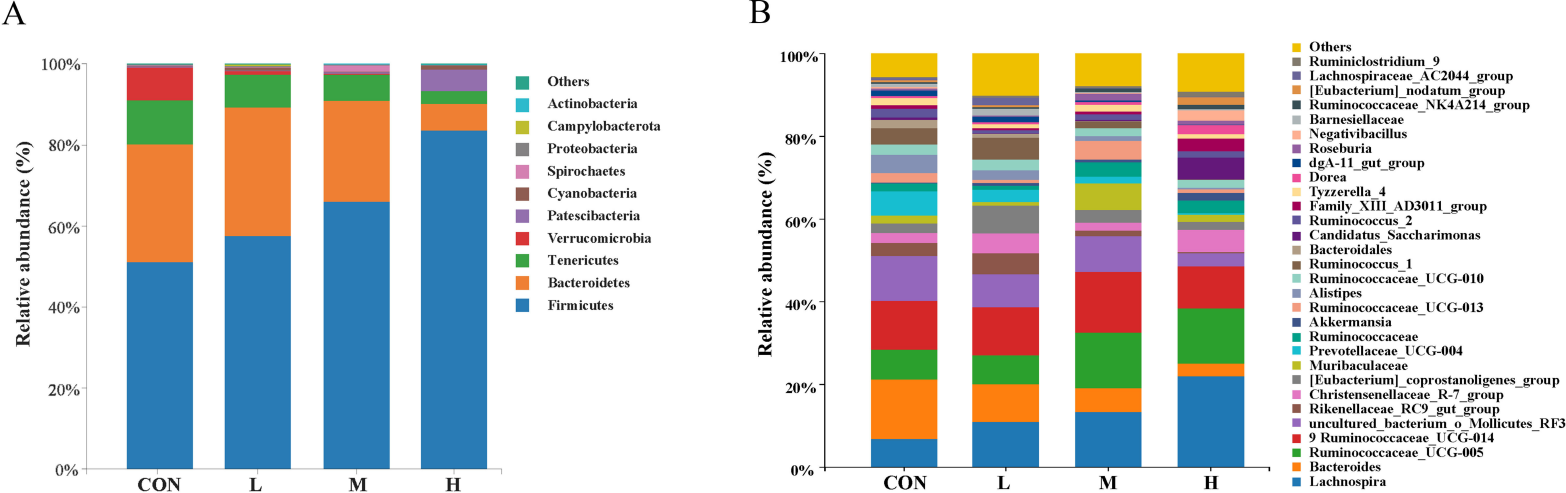
Changes in fecal microbiota at the phylum (**A**) and genus (**B**) levels in growing phase FMD-fed diets with different digestible energy levels.

At the phylum level, the fecal microbiota in the growing phase FMD was dominated by *Firmicutes* and *Bacteroidetes*. As the dietary energy level increased, the relative abundance of *Firmicutes* increased, while the relative abundance of *Bacteroidetes* and *Tenericutes* decreased. *Verrucomicrobia* and *Patescibacteria* were among the dominant phyla in the CON and H groups, respectively (Fig. 4A). Figure 4B shows the top 30 genera with the highest relative abundance at the genus level, with *Lachnospira* increasing with higher dietary energy levels and *Bacteroides* exhibiting the opposite trend.

#### Beta diversity analysis based on OTUs

**Fig 5:**
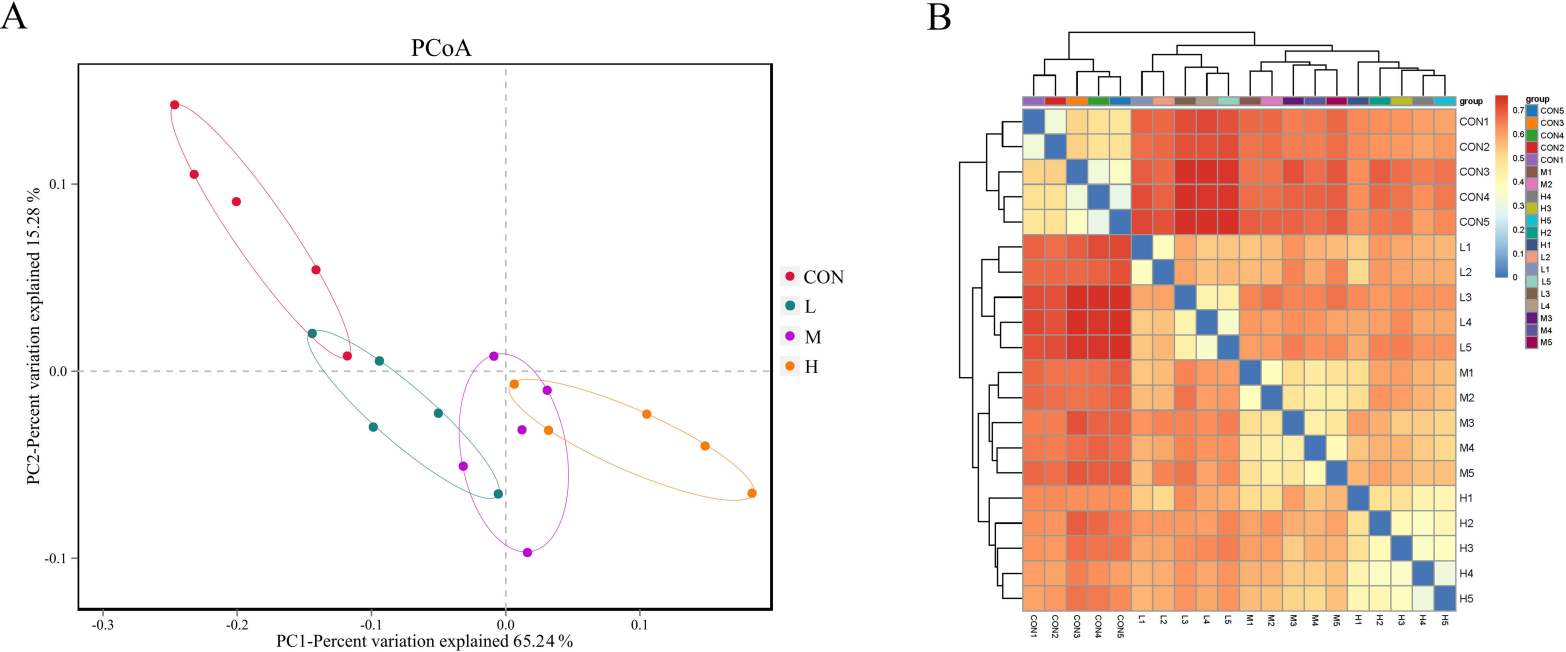
Principal coordinates analysis (PCoA) (**A**) and heatmap of sample distances (**B**) of fecal microbiota in growing phase FMD-fed diets with different digestible energy levels. Red indicates the closest distances, while blue indicates longer sample-to-sample distances.

Principal coordinates analysis (PCoA) showed that the CON group was clustered farther from the other three pellet-fed groups (Fig. 5A). A heatmap of sample distances indicated that replicates within each treatment clustered together, with the CON and L groups closer in proximity compared to the other groups, and the CON group being farthest from the H group (Fig. 5B).

#### Group differences and microbiota correlation analysis

**Fig 6:**
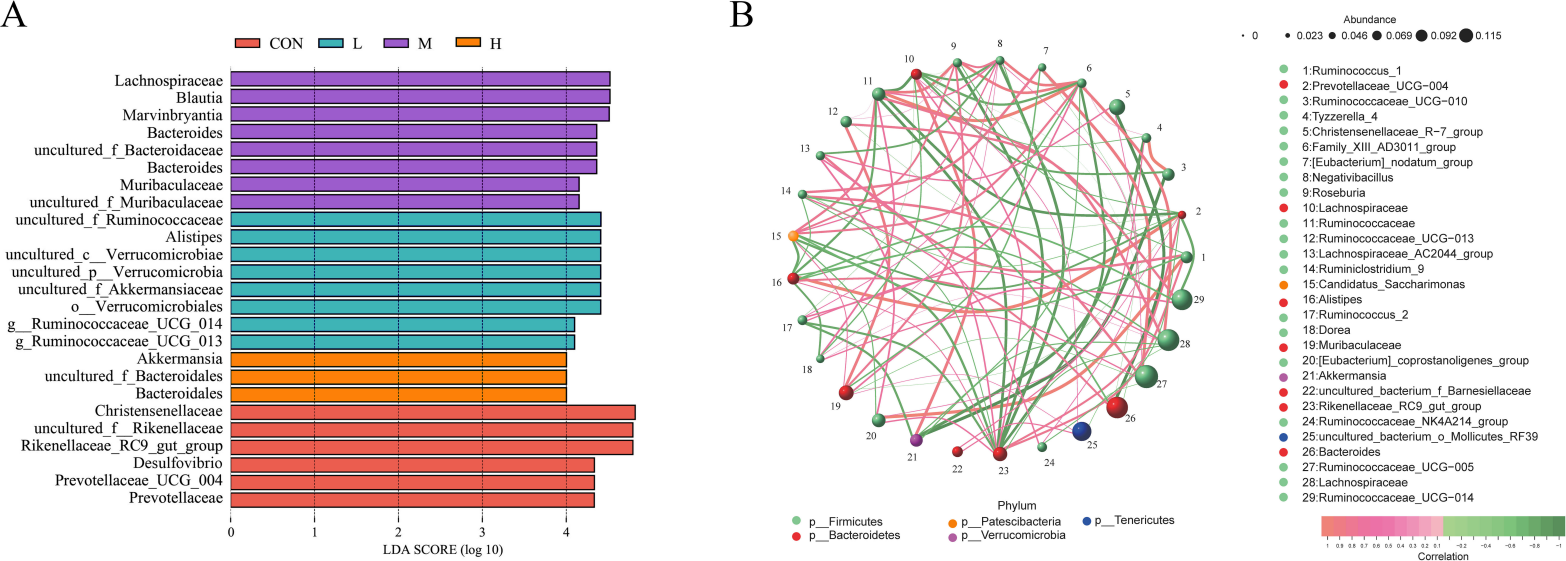
Linear discriminant analysis effect size, LEfSe analysis of group differences (**A**), and microbiota correlation analysis (**B**) of fecal microbiota in growing phase FMD-fed diets with different digestible energy levels.

To further investigate the characteristics of the fecal microbiota in different treatment groups, LEfSe analysis was performed. It revealed that *Prevotellaceae*, *Desulfovibrio*, and *Rikenellaceae* were significantly enriched in the CON group, while *Bacteroidales* and *Akkermansia* were significantly enriched in the H group (Fig. 6A). Correlation analysis in Fig. 6B showed that *Prevotellaceae_UCG-004* was positively correlated with *Alistipes*, and *Rikenellaceae_RC9_gut_group* was positively correlated with *Ruminococcus_1* and *Prevotellaceae_UCG-004* but negatively correlated with *Ruminococcaceae*, *Roseburia*, and *Negativibacillus. Akkermansia* was positively correlated with *Lachnospira* and negatively correlated with *Ruminococcaceae_UCG-010* and *Tyzzerella_4*.

## DISCUSSION

Intestinal diseases, particularly diarrhea, have become one of the main limiting factors in the rapid development of FMD farming (17–19). In our study, the slight increase in diarrhea rate observed in the first 3 weeks among the pellet-fed groups could be attributed to the adaptation period required for FMD to adjust to the new feed and feeding method after transitioning from traditional feeding to a completely mixed pelleted diet (20, 21). In the middle and later stages of the experiment, as well as throughout the entire trial period, the diarrhea rate in the pellet-fed groups (L, M, and H) was lower than in the traditional feeding group (CON), indicating the safety of the feed used in our study.

In further studies on the growth performance of FMD, the ADFI (total weight) of the CON group was more than two times that of any other group. This was primarily due to the high palatability of carrots and pumpkins, which are commonly used in traditional feeding practices, leading the musk deer to consume large amounts of these succulent feeds. However, succulent feeds have a high water content and a strong satiety effect, resulting in a significantly lower average daily dry matter intake in the CON group compared to the other three groups. Since the gastrointestinal tract of animals is limited by its volume and digestion rate, feed intake capacity is constrained (22). Consequently, although the DE of the CON group feed in its dried form was equivalent to that of the M group, the actual energy intake by the animals in the CON group was much lower. Similarly, this finding indicates that the energy level of 8.87 MJ/kg in the L group was insufficient to meet the nutritional requirements of musk deer at this stage. In terms of ADG, shoulder height, body length, and body oblique length, the H group significantly outperformed the other three groups, suggesting that the energy level of this diet was more suitable for growing phase FMD. Although the DE content in the CON group was similar to that of the M group, the growth performance and body measurement increases in the M group were significantly higher, likely due to the low dry matter intake in the CON group, which could not meet the energy and nutrient requirements of growing phase FMD. Research reported that excessively low dietary energy levels hinder lamb growth performance and rumen development (23).

In our study, the digestibility of nutrients was higher in the high DE groups compared to the low DE groups, explaining the superior growth performance of the H group.

The diversity and stability of the gut microbiota are key factors in maintaining health resilience (24). In our study, FMD fed a high-digestible-energy diet exhibited the best growth performance and the highest fecal microbiota alpha diversity index, further confirming the important role of gut microbial diversity in intestinal health (25, 26). In addition to alpha diversity, beta diversity of the gut microbiota also reflects the host’s physiological status and health (27). Studies have shown that the gut microbiota is influenced by dietary DE levels (28, 29), which may explain why the clustering of the CON group was more similar to the group fed a low DE diet. Additionally, diet composition is one of the most significant factors affecting the gut microbiota of animals (30). In our study, compared to the CON group, the traditional feeding regimen for FMD included large amounts of succulent feeds, such as carrots, which differ substantially from the ingredients used in pelleted feed. Research by Forwood et al. (31) demonstrated that adding carrots to goat feed altered the rumen microbiota structure and influenced tyrosine degradation pathways, which was suggested to improve feed conversion efficiency in goats consuming carrots (31). This may also explain why, in our study, the CON group had a similar DE intake to the L group but exhibited a lower feed-to-gain ratio (F/G).

Our results showed that *Firmicutes* and *Bacteroidetes* were the dominant phyla in the fecal microbiota of all four groups, consistent with previous reports (2, 17, 32, 33). Additionally, the *Firmicutes/Bacteroidetes* ratio exhibited an increasing trend with higher dietary energy levels. Studies have shown that an elevated *Firmicutes/Bacteroidetes* ratio is positively correlated with weight gain in animals (34, 35). *Verrucomicrobia* was one of the unique dominant phyla in the CON group. Studies have shown that a reduction in *Verrucomicrobia* may positively influence rumen fermentation and contribute to methane reduction (36). Methane production during rumen fermentation represents a major pathway for energy loss in ruminants (37).

At the genus level, the relative abundance of *Lachnospira* gradually increased from the CON group to the H group. *Lachnospira* has been reported to produce beneficial metabolites, such as butyrate, which plays a critical role in repairing intestinal epithelial cells (38). Supporting this, the study by Guo et al. (39) demonstrated that *Lachnospira* generates substantial amounts of short-chain fatty acids, with its relative abundance showing a significant negative correlation with the duration of diarrhea in patients (39). This finding provides a plausible explanation for the lowest diarrhea rate observed in the H group.

Further LEfSe analysis revealed that *Desulfovibrio* and *Akkermansia* were among the significantly differentiated genera in the CON group. *Desulfovibrio* is a major producer of hydrogen sulfide (H_₂_S) in the gastrointestinal tract (40), and H_₂_S has been associated with chronic colonic diseases and intestinal inflammation (41). As a probiotic in the gut, *Akkermansia* utilizes host-secreted mucin to colonize the gut through competitive exclusion, protecting the intestine from pathogen invasion (42).

Network analysis further indicated a strong positive correlation between the relative abundance of *Akkermansia* and *Lachnospira*, while *Akkermansia* exhibited a negative correlation with *Tyzzerella_4*. Previous research has shown that, in humans, *Tyzzerella_4* is positively associated with the risk of ileal Crohn’s disease (43).

In summary, our study suggests that pellet feed can reduce the diarrhea rate and improve feed efficiency during the rearing period of FMD, as evidenced by microbial community changes.

### Conclusion

The results of this study indicate that the total mixed pelleted diet used in this trial is suitable for the rearing of growing phase FMD, and that at a DE level of 11.86 MJ/kg, FMD exhibited optimal growth performance, the highest nutrient digestibility, and the greatest diversity of gut microbiota, with the highest relative abundances of probiotic genera such as *Akkermansia* and *Lachnospira*.

## Data Availability

The 16S rRNA sequencing data sets have been uploaded to the Sequence Read Archive (SRA) database under BioProject number PRJNA1182763.
